# Exploring health insurance and knowledge of the ovulatory cycle: evidence from Demographic and Health Surveys of 29 countries in Sub-Saharan Africa

**DOI:** 10.1186/s12978-023-01675-z

**Published:** 2023-08-30

**Authors:** Betregiorgis Zegeye, Dina Idriss-Wheeler, Olanrewaju Oladimeji, Sanni Yaya

**Affiliations:** 1 HaSET Maternal and Child Health Research Program, Addis Ababa, Ethiopia; 2 Interdisciplinary School of Health Sciences, University of Ottawa, Ottawa, Canada; 3 Faculty of Health Sciences, Department of Public Health, Walter Sisulu University, Eastern Cape, Mthatha, South Africa; 4 School of International Development and Global Studies, University of Ottawa, Ottawa, Canada; 5 The George Institute for Global Health, Imperial College London, London, UK

**Keywords:** Ovulatory cycle knowledge, Women’s health, Health insurance, Sub-Sahara Africa, DHS, Global health

## Abstract

**Background:**

Unplanned pregnancy continues to be a major public health concern in Sub-Saharan Africa (SSA). Understanding the ovulatory cycle can help women avoid unplanned pregnancy. Though a wide range of factors for ovulatory cycle knowledge in SSA countries has not been well assessed, the influence of health insurance on ovulatory cycle knowledge is largely unknown. As a result, we set out to investigate the relationship between health insurance enrollment and knowledge of the ovulatory cycle among women of childbearing age. This study aims to investigate the relationship between health insurance enrollment and knowledge of the ovulatory cycle among women of childbearing age in sub-Saharan Africa (SSA).

**Methods:**

Demographic and Health Surveys (DHSs) data from 29 SSA countries were analyzed. The association between health insurance and ovulatory cycle knowledge was investigated using bivariate and multivariate multilevel logistic regression models among 372,692 women of reproductive age (15–49). The findings were presented as adjusted odds ratios (AOR) with 95% confidence intervals (CI). A p-value of 0.05 was considered statistically significant.

**Results:**

The pooled result shows that the prevalence of knowledge of ovulatory cycle in the studied 29 SSA countries was 25.5% (95% CI; 24.4%-26.6%). Findings suggest higher odds of ovulatory cycle knowledge among women covered by health insurance (AOR = 1.27, 95% CI; 1.02–1.57), with higher education (higher-AOR = 2.83, 95% CI; 1.95–4.09), from the richest wealth quintile (richest-AOR = 1.39, 95% CI; 1.04–1.87), and from female headed households (AOR = 1.16, 95% CI; 1.01–1.33) compared to women who had no formal education, were from the poorest wealth quintile and belonged to male headed households, respectively. We found lower odds of ovulatory cycle knowledge among women who had 2–4 parity history (AOR = 0.80, 95% CI; 0.65–0.99) compared to those with history of one parity.

**Conclusions:**

The findings indicate that the knowledge of the ovulatory cycle is lacking in SSA. Improving health insurance enrollment should be considered to increase ovulatory cycle knowledge as an approach to reduce the region's unplanned pregnancy rate. Strategies for improving opportunities that contribute to women’s empowerment and autonomy as well as sexual and reproductive health approaches targeting women who are in poorest quintiles, not formally educated, belonging to male headed households, and having high parity should be considered.

## Background

Fertility awareness is knowing the likelihood of pregnancy during the menstrual cycle [1], being aware of the risks associated with sexual activity, and making decisions about conception for women who are engaging in sexual activity [2]. As a result, knowledge and understanding of a woman's ovulatory cycle is an essential component of fertility awareness for successful pregnancy and family planning [3]. Ovulation is the physiological event when the egg is released from the ovary for potential fertilization—it is the fertile phase of the menstrual cycle during which the chances of pregnancy are greatest [3–5]. Indeed, evidence suggests that sexual and reproductive health (SRH) knowledge (i.e., the female reproductive system, fertility awareness, ovulatory cycle) among women and adolescents tends to be limited [6, 7], leading to unmet family planning needs such as unintended and unwanted pregnancies. According to existing literature, women tend to have a poor understanding of their ovulation cycle. For example, in the United States, 33% of women had correct knowledge of their ovulatory cycle [7], whereas in Spain and India, this rate was around 31% [8] and 15%, respectively [9]. In the Africa region, Ethiopia (23%) [10], Kenya (23%), Gambia (23%), Guinea (23%), Sao Tome and Principe (10%), Namibia (14%), Nigeria (20.%), Zambia (22%), and Rwanda (21. %) reported low prevalence of ovulation cycle knowledge [2]. Consequently, a better understanding of ovulation and fertility may help reduce the incidence of unintended pregnancy and associated negative health outcomes, particularly in Sub-Saharan Africa (SSA) [11–14].

Bearack et al. found that, globally, 44% of pregnancies among women aged 15–44 years old were unintended [15], the burden of which lies in low- and middle- income countries (LMICs). In sub-Saharan Africa (SSA), one study suggested close to 20 million unintended pregnancies take place annually [16] and another estimated a 29% prevalence of unintended pregnancies [16]. A recently published study on determinants of knowledge of ovulatory cycle (KOC) across 29 countries in SSA revealed correct KOC among women was a mere 15.5% [17]. Incidentally, uptake of modern contraceptives (MC) in the region is also low, an estimated 22% in SSA [18]. Studied barriers include lack of provider, availability (i.e., stock), lack of satisfaction with method available, perceived negative attitudes of health workers, cultural norms and a climate of fear around modern contraception use in the SSA region [19–21]. A recent systematic review detailed additional reasons for non-use of modern family planning methods that included “fear of side effects, husband’s disapproval, the absence of menses, abstinence, and low perception of risk of pregnancy.” [22]. With low modern contraception uptake and increased likelihood of negative health and social consequences of unintended pregnancies, the importance of reinforcing the understanding and knowledge of natural family planning methods—SRH literacy, fertility awareness, KOC—is emphasized.

The lack of SRH knowledge, literacy, and awareness coupled with low use of modern contraception contributes to negative outcomes such as unwanted or unplanned pregnancies and unsafe abortions, particularly among adolescents and marginalized groups [7, 23–25]. Adolescent females are at higher risk of unplanned pregnancies and are more likely to be diagnosed with anemia, have complications and to die during pregnancy [26]. Women who have unintended pregnancies are 20–22% more likely to develop maternal depression and score higher on the parenting stress score [27]. Of the estimated 62.5 million pregnancies in Africa between 2015 and 2019, 43% were unintended, of which, 40% ended in an abortion [28]. With restricted abortion laws, poor quality health services and existing social norms around abortions and unwed mothers, maternal mortality rates due to unsafe abortions remain high in SSA [29]. Although contraception can be considered a comprehensive family planning strategy, its effectiveness as an approach has been called into question with the rise of unintended pregnancy rates and known low uptake in LMICs [16, 30, 31]. As an alternative method to modern family planning, a fertility awareness (i.e., natural family planning) approach such as knowledge of ovulatory cycle (KOC) is being proposed [32].

A recent study investigated the individual and community level determinants of KOC in SSA to inform appropriate strategies to increase knowledge and reduce unwanted or unplanned pregnancies [17]. Knowledge of ovulatory cycle among women of reproductive age was found to be low in the region and varied by country. Women’s age and educational level were the individual-level factors associated with increased knowledge of ovulatory cycle. Several studies have shown the higher likelihood of KOC among women who are educated and wealthy, leading to better SRH outcomes. This highlights the inequities of access to care for socioeconomically disadvantaged women and emphasizes the UN’s Sustainable Development Goal 3.8 call to action for universal health coverage (UHC) for all [33].

It is well documented that out-of-pocket payments are the leading approach, and subsequently, a deterrent to accessing health services in SSA [34]. This impacts sub-groups—particularly women and adolescents—who do not have the financial means or decision-making autonomy to seek or access healthcare, resulting in negative social and health outcome [35]. Health insurance schemes—established systems that reduce financial risk of healthcare expenditures—are the vehicles used by governments globally to work towards UHC [36]. There is evidence to suggest that having health insurance is a positive factor in increasing health-related knowledge and seeking of maternal and child health services [37–39]. Additionally, both women’s empowerment [40] and household decision-making [41] have been shown to have a positive association with health insurance coverage, suggesting possible opportunities to further investigate women’s health seeking and access to SRH knowledge, awareness and services. Yet the gap between health insurance and knowledge of ovulatory cycle remains unknown. As a result, the purpose of this study was to investigate the relationship between health insurance and knowledge of the ovulation cycle in 29 SSA countries. Findings will support the development of specific reproductive health interventions to ensure appropriate ovulatory cycle knowledge among women and in communities. This can help women's health by preventing unwanted pregnancies and associated social and health inequities, especially for those who have limited access to and knowledge of contraceptive methods.

## Methods

### Data source

Data from 29 nationally representative Demographic Health Surveys (DHS) in SSA were extracted for this study. The DHS Program works closely with national organization in LMICs to collect and monitor demographic and health indicators that tend to be used to inform policy and evidence-based decision making [42]. Included in these demographic surveys are key sexual and reproductive health indicators such as health insurance coverage and knowledge of ovulatory cycle. United States Aids for International Development (USAID) and International Child Fund (ICF) provide financial and technical support to LMICs based on localized need for data collection [43].

For all 29 SSA countries included, a stratified two-stage cluster sampling method was used. In the first stage, Enumeration Areas (EA) was systematically selected from the sampling frame prepared for the most recent and available national population census Subsequently, in the second stage, Probability Proportional to Size (PPS) is used to select a fixed number of households (typically 25–30) [44]. The Individual Recode (IR) file, which was publicly available through DHS Program (https://dhsprogram.com/data/available-datasets.cfm) was used for analysis.

### Inclusion criteria

A total of 29 countries were included based on the following inclusion criteria: country with latest DHS data available to download between 2010 and 2020, availability of variables of interest (outcome and explanatory), country is located geographically in SSA. Table 1 outlines the year of survey and weighted sample in each included country.

**Table 1:** Year of survey and weighted sample in each studied country

Country	Year of survey	Sampled population	Weighted %
Angola	2015/16	14,379	3.9
Burkina Faso	2010	17,087	4.5
Benin	2017/18	15,928	4.3
Burundi	2016/17	17,269	4.6
Chad	2014/15	17,580	4.7
Congo Democratic Republic	2013/14	18,802	5.0
Congo	2011/12	10,819	2.9
Cote d’Ivoire	2011/12	10,047	2.7
Cameroon	2018/19	13,527	3.6
Ethiopia	2016	15,683	4.2
Gabon	2012	8,422	2.3
Ghana	2014	9,395	2.5
Gambia	2019/20	10,172	2.7
Guinea	2018	10,874	2.9
Comoros	2012	5,329	1.4
Kenya	2014	14,724	4.0
Liberia	2019/20	9,239	2.5
Lesotho	2014	6,621	1.8
Mali	2018	10,519	2.8
Malawi	2015/16	24,562	6.6
Niger	2012	11,160	3.0
Namibia	2013	9,166	2.5
Sierra Leone	2019	15,574	4.2
Senegal	2010/11	15,688	4.2
Togo	2013/14	9,468	2.5
Uganda	2016	18,506	5.0
South Africa	2016	8,514	2.3
Zambia	2018/19	13,683	3.7
Zimbabwe	2015	9,955	2.7
Total		372,692	100.0

### Outcome variable

The outcome variable in this study was women’s correct knowledge of ovulatory cycle. Respondents to the survey were asked, "when is the ovulation time?" and provided with the following six possible response options: "during her period", "after period ended", "middle of the cycle", "before the period begins", "at any time", and "don’t know". This variable was recoded into a binary (dichotomized) variable to be able to complete a regression analysis. If a participate answered correctly—ovulation takes place in “middle of the cycle”—they were coded as '1’; all other responses were incorrect and coded as ‘0’ [10, 45, 46].

### Explanatory variables

The key explanatory variable of interest was coverage by health insurance at the time of the survey. Participants were asked if they had either public or private insurance (i.e., social security, employer-based insurance, mutual health organization or community-based insurance, privately purchased commercial insurance). If they indicated they were covered by either public or private insurance, they were coded as “yes”, if they indicated they were not covered by any health insurance, they were coded as “No” [46, 47].

On the basis of epidemiological knowledge and prior evidence [1, 2, 7–10, 23], we identified potential demographic, socioeconomic and obstetric confounding factors to consider in the model: women’s age (categories: 15–19, 20–24, 25–29, 30–34, 35–39, 40–44, 45–49), women’s and husband’s educational level (no formal education, primary school, secondary school, higher)), wealth index (poorest, poorer, middle, richer, richest), currently employed (yes, no), marital status (not married, married), head of household (male, female), parity (one birth, 2–4 births, five and above births), place of residence (urban, rural), distance to health facility (big problem, not a big problem) and media exposure (yes, no). For media exposure, yes was for women who indicated they had at least read a newspaper and/or watched television and/or listened to radio at least once a week; no was if the women indicated they had not accessed any of the three media listed—newspaper, television, or radio—at least once a week.

### Statistical analyses

Stata version 14 software was used for the analysis. First, we completed descriptive analysis (i.e., frequencies) of the knowledge of ovulatory cycle (outcome variable) along with the explanatory and confounding variables we were considering for our model; these were presented using tables and graphs. This was followed by a bivariate logistic regression analysis to select candidate control variables at p-value less than 0.05 (p ≤ 0.05). A multicollinearity test using the variance inflation factor (VIF)—Mean VIF = 2.46, Min VIF = 1.03, Max VIF = 5.42—revealed no evidence of collinearity as a VIF less than 10 is tolerable [48]. A multivariate logistic regression analysis was conducted including all explanatory variables that were selected at bivariate analysis, to investigate the association between health insurance coverage and knowledge of ovulatory cycle. Using the Hosmer–Lemeshow test confirmed that the model was fit (p = 0.578). The logistic regression model results were presented using Crude Odds Ratio (COR) and Adjusted Odds Ratio (AOR) at the 95% Confidence Interval (CI). The “svyset” command was used to consider weight, cluster and strata in DHS’s data sampling methods. The Strengthening of Observational Studies in Epidemiology (STROBE) guidelines were used in preparation of this manuscript [49].

### Ethical considerations

This study uses secondary data analysis to quantitively investigate publicly available DHS data and therefore no ethical approval was required to conduct this work (https://dhsprogram.com/data/available-datasets.cfm). Ethical data collection methods and approaches were considered during participant recruitment and survey dissemination by the institutions that funded, commissioned, and managed the surveys. All the DHS surveys used in this study were approved by ICF international and followed the U.S. Department of Health and Human Services rules for respecting of human subject’s rights (refer to http://goo.gl/ny8T6X for more information on protecting the privacy of DHS participants).

## Results

### Description of study population

A total of 372,692 women of reproductive age (15–49 years) participated in this study. Around 37.0% of those surveyed were between the ages of 15 and 24. Around 59.5% and 22.7% of respondents were rural residents and reported having a difficult time visiting health facilities, respectively. About half of the respondents (50.2%) attended primary school, while 6.9% did not receive formal education. Almost two-fifths (38.5%) of those polled were unemployed (Table 2).

**Table 2:** Background characteristics of respondents and distribution of ovulatory cycle knowledge across explanatory variables: evidence from 29 SSA countries’ DHS

Variable	Frequency (weighted %)	Ovulatory cycle knowledge (weighted %)	Chi-square (χ^2^)	*p*-value (*p*)
Health insurance			280.65	< 0.001
No	329,549 (81.9)	22.7		
Yes	28,312 (18.1)	38.3		
Women’s age			123.42	< 0.001
15–19	80,142 (18.6)	17.3		
20–24	69,326 (18.4)	27.8		
25–29	64,991 (20.0)	27.2		
30–34	52,794 (14.8)	26.3		
35–39	44,861 (12.2)	28.5		
40–44	33,477 (8.8)	26.0		
45–49	27,101 (7.2)	28.9		
Women’s educational level			623.64	< 0.001
No formal education	127,972 (6.9)	16.9		
Primary school	112,578 (50.2)	19.9		
Secondary school	116,867 (32.1)	28.4		
Higher	15,231 (10.8)	48.5		
Husband educational level			222.85	< 0.001
No formal education	93,053 (6.8)	20.4		
Primary school	60,713 (46.9)	20.8		
Secondary school	71,929 (32.8)	29.0		
Higher	17,500 (13.4)	39.1		
Economic status			295.55	< 0.001
Poorest	77,059 (15.3)	17.7		
Poorer	71,893 (17.7)	20.9		
Middle	71,386 (19.6)	22.4		
Richer	71,735 (21.3)	26.3		
Richest	80,619 (26.2)	34.9		
Parity			43.23	< 0.001
One	53,255 (21.6)	30.4		
2–4	127,191 (53.0)	25.8		
5 +	93,771 (25.5)	22.4		
Marital status			8.98	< 0.0539
Not married	181,072 (45.5)	24.3		
Married	191,620 (54.5)	26.5		
Household head			11.65	< 0.0117
Male	266,231 (61.2)	22.8		
Female	106,461 (38.8)	27.2		
Currently employed			34.21	< 0.001
No	160,254 (38.5)	22.8		
Yes	211,984 (61.5)	27.2		
Media exposure			29.75	< 0.001
No	120,823 (13.4)	19.7		
Yes	238,182 (86.6)	27.1		
Place of residence			82.59	< 0.001
Urban	141,814 (40.5)	29.5		
Rural	230,878 (59.5)	22.8		
Distance to health facility			43.36	< 0.001
Big problem	143,230 (22.7)	21.1		
Not a big problem	213,593 (77.3)	26.8		

### Coverage of health insurance

**Fig. 1:**
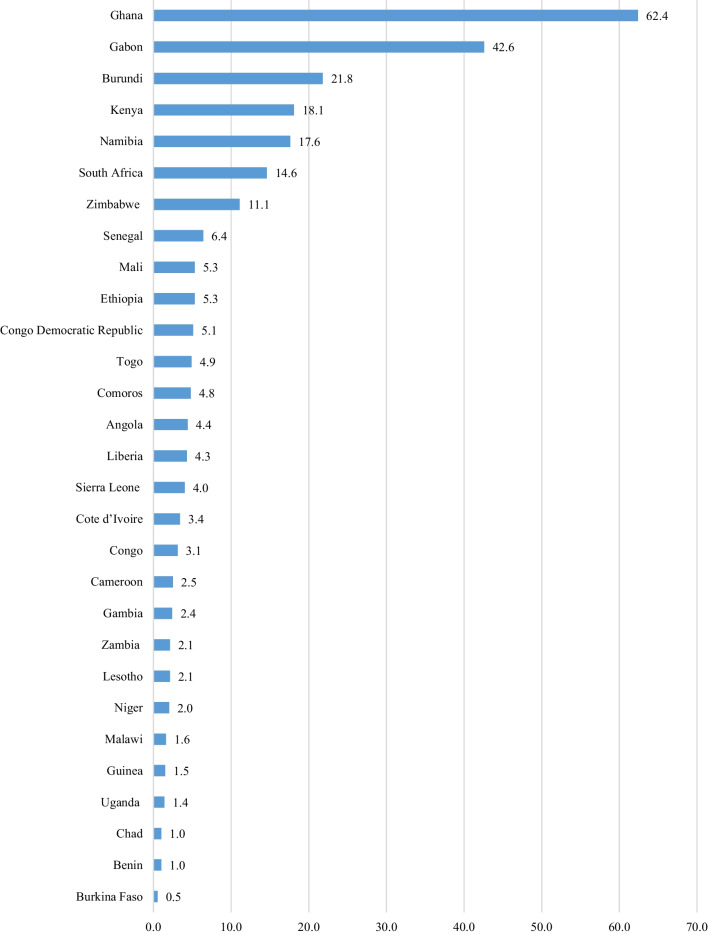
Coverage of health insurance among women of reproductive age (15–49 years) across 29 SSA countries

In the pooled data, approximately 18.1% of women of reproductive age had health insurance. Ghana (62.4%), Gabon (42.6%), and Burundi (21.8%) had the highest coverage, while Burkina Faso (0.5%), Benin (1.0%), and Chad (1.0%) had the lowest coverage (Fig. 1).

### Prevalence of ovulatory cycle knowledge

**Fig. 2:**
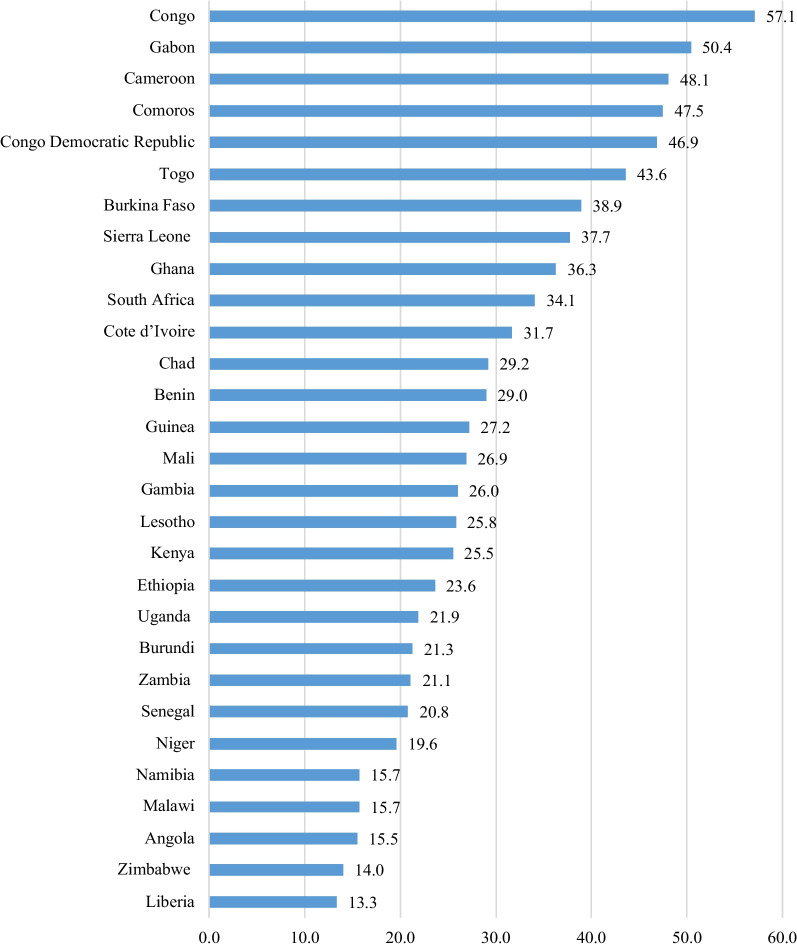
Prevalence of ovulatory cycle knowledge among women of reproductive age (15–49 years) across 29 SSA countries

The pooled result shows about 25.5% of women in the reproductive age had correct ovulatory cycle knowledge. The highest prevalence was in Congo (57.1%), Gabon (50.4%) and Cameroon (48.1%), while lowest prevalence was seen in Liberia (13.3%), Zimbabwe (14.0%) and Angola (15.5%) (Fig. 2).

### Distribution of ovulatory cycle knowledge across explanatory and confounding variables

The variation in ovulatory cycle knowledge across the explanatory variable, confounding variables and subgroups is shown in Table 2. Knowledge of the ovulatory cycle ranged from 22.7% among women without health insurance to 38.3% among women with health insurance. Additionally, there was a 31.6% difference in ovulatory cycle knowledge between women who did not attend formal education (16.9%) and women who did (48.5%). Ovulatory cycle knowledge ranged from 17.7% for women from the poorest households to 34.9% for women from the richest households, and from 19.7% among women who had been exposed to media, to 27.1% among women who had not been exposed to media (Table 2).

### Health insurance as a predictor of ovulatory cycle knowledge

As shown in Table 3, women with health insurance had a higher likelihood of knowing their ovulatory cycle (AOR = 1.27, 95% CI; 1.02–1.57) than women without health insurance. Furthermore, women who attended secondary school (AOR = 1.72, 95% CI; 1.29–2.31) or higher (AOR = 2.83, 95% CI; 1.95–4.09) were more likely to know the ovulatory cycle than women who did not attend formal education. The results also show that women from the richest households have a higher likelihood of knowing about the ovulatory cycle (AOR = 1.39, 95% CI; 1.04–1.87) than women from the poorest households. Women from female-headed households had a higher likelihood of knowing about the ovulatory cycle (AOR = 1.16, 95% CI; 1.01–1.33) than women from male-headed households. However, women with higher parity (2–4 births) had lower odds of ovulatory cycle knowledge (AOR = 0.80, 95% CI; 0.65–0.99) than those with one parity.

**Table 3:** Bivariate and multivariable logistic regression results for association between health insurance and ovulatory cycle knowledge among reproductive age women in SSA: evidence from 29 SSA countries’ DHS

Variable	COR (95% CI)	AOR (95% CI)
Health insurance		
No	Ref.	Ref.
Yes	2.11 (1.83–2.44)***	1.27 (1.02–1.57)*
Women’s age		
15–19	Ref.	Ref.
20–24	1.83 (1.55–2.16) ***	0.80 (0.51–1.28)
25–29	1.78 (1.48–2.13) ***	0.99 (0.62–1.60)
30–34	1.70 (1.42–2.04) ***	0.96 (0.60–1.55)
35–39	1.89 (1.58–2.27) ***	1.16 (0.72–1.86)
40–44	1.67 (1.36–2.05) ***	1.17 (0.70–1.96)
45–49	1.93 (1.57–2.38) ***	1.21 (0.72–2.03)
Women’s educational level		
No formal education	Ref.	Ref.
Primary school	1.22 (1.02–1.46) *	1.26 (0.98–1.63)
Secondary school	1.94 (1.61–2.35) ***	1.72 (1.29–2.31)***
Higher	4.63 (3.63–5.91) ***	2.83 (1.95–4.09)***
Husband educational level		
No formal education	Ref.	Ref.
Primary school	1.02 (0.83–1.26)	0.78 (0.58–1.04)
Secondary school	1.59 (1.27–1.98) ***	0.95 (0.69–1.32)
Higher	2.49 (1.94–3.19) ***	0.87 (0.60–1.25)
Economic status		
Poorest	Ref.	Ref.
Poorer	1.22 (1.04–1.43) *	0.98 (0.78–1.23)
Middle	1.34 (1.14–1.58) ***	0.97 (0.76–1.23)
Richer	1.66 (1.41–1.95) ***	1.12 (0.87–1.44)
Richest	2.49 (2.09–2.97) ***	1.39 (1.04–1.87)*
Parity		
One	Ref.	Ref.
2–4	0.79 (0.68–0.92) **	0.80 (0.65–0.99)*
5 +	0.66 (0.55–0.78) ***	0.76 (0.57- 1.00)
Marital status		
Not married	Ref.	Ref.
Married	1.12 (0.99–1.25)	NA
Household head		
Male	Ref.	Ref.
Female	1.14 (1.02–1.26) *	1.16 (1.01–1.33)*
Currently employed		
No	Ref.	Ref.
Yes	1.25 (1.12–1.40)***	0.99 (0.85–1.15)
Media exposure		
No	Ref.	Ref.
Yes	1.51 (1.27–1.80) ***	1.00 (0.80–1.24)
Place of residence		
Urban	Ref.	Ref.
Rural	0.70 (0.62–0.79) ***	1.10 (0.93–1.30)
Distance to health facility		
Big problem	Ref.	Ref.
Not a big problem	1.36 (1.21–1.53) ***	1.03 (0.88–1.21)

*p < 0.05; **p < 0.01; ***p < 0.001; Ref. = reference category

## Discussion

The association between health insurance and knowledge of the ovulatory cycle among reproductive-aged women was investigated in this study using nationally representative data from 29 SSA countries. The pooled results show that approximately 25.5% (95% CI; 24.4%–26.6%) of reproductive-age women were aware of the ovulatory cycle. This finding is lower than a previous study among young women in SSA, which found that 49% of young women had correct ovulation knowledge [2]. This could be because the previous study only included young women (15–24 years) [2] while our study included all women of reproductive age (15–49 years), and young women tend to have better health knowledge [50]. Additionally, there was a difference in prevalence of knowledge of the ovulatory cycle observed among the 29 SSA countries. One possible explanation for the variation within SSA is the countries’ health literacy levels. Evidence suggests that health literacy can be effective and improve population level awareness [51]. Some low and middle income countries (LMICs) have implemented innovative health literacy approaches and demonstrated exemplar outcomes that improved health related knowledge, however, these are varied and depend on local or national government investments [51], which are at the will of political powers. Additionally, health literacy in Africa is positively associated with higher education, wealth, and living in urban areas [52]. Sub-Saharan African countries will accordingly vary in their national or local education, wealth status and population living in urban versus rural regions, all of which influence and explain differing population health literacy levels across countries in the region.

Findings revealed that women with health insurance were more likely to know about the ovulatory cycle than women with no health insurance. There is little evidence to support this finding, however evidence exists on lower health literacy being associated with avoiding seeking health services [53]. Women with health insurance tend to have more contacts with health services and health personnel, and more likely to be exposed to reproductive health information and education [53]. Education and wealth status have been shown to be positively associated with having health insurance coverage in SSA [54, 55] which are also highly correlated with health literacy [52]. Evidence suggests that children, adolescents, women, and other family members in insured households are more likely to receive health services, be informed about health-related knowledge, and are healthier than those in uninsured households [37]. Several studies have also found that women and children who are uninsured or have inadequate health insurance coverage, are less likely to visit health facilities and access health information, preventive, curative, and other care and support health services [38, 56]. Although education, economic, social, cultural, and geographical barriers to healthcare must be addressed, affordable health insurance can be part of the solution as countries work towards universal health coverage [57, 58].

In this study, women's educational level was related to their knowledge of the ovulatory cycle. This finding was consistent with a systematic review conducted by Pedro et al. [59] and studies conducted in Uganda [60] and Ethiopia [61]. It is possible that higher education allows students to learn more about the physiology of reproduction and health related knowledge [10]. Various educational approaches aimed at increasing fertility awareness are currently being developed and researched. For example, a pre-post interventional study in Canada found that fertility education websites can raise a person’s fertility awareness [62]. Another study in Rwanda found that an entertainment-education serial radio drama raised fertility awareness [63].

Similar to a previous study in Ethiopia, women from the richest households had a higher likelihood of knowing about the ovulatory cycle than women from the poorest households [10]. As Tucker-Drob and Briley suggest, adolescents belonging to higher socioeconomic status (SES) (wealthier quintiles) can pursue interest and knowledge as compared to individuals who are of lower SES, restricting their knowledge base [64].

Women from female-headed households (FHH) had higher odds of knowing about the ovulatory cycle than women from male-headed households (MHH). There are several factors that influence effective communication of sexual and reproductive health issues between parents and their adolescents in Africa. Gender, religious and cultural norms exist that are barriers to communication, particularly between male parents and their daughters [65]. Reasons for these barriers include embarrassment of the topic, taboo and unaccepted topic, cultural and religious beliefs around sexual health or thinking adolescents are too young to understand the concepts, they lead to sexual and reproductive health information and education gaps [65] A recent scoping review by Agbeve et al., detailed the gendered trends of sexual and reproductive health communication between adolescents and their parents. They highlighting that mothers preferred discussing SRH topics with their daughters, while fathers with their sons, further indicating that mothers, more than fathers, tended to be the “sex educator” for both genders, and cultural norms dictated that fathers were not expected to communicate with their daughters on sexuality related issues [66]. This explains the increased likelihood that FHH had higher odds of knowing about the ovulatory cycle than MHH. Additionally, several studies have found that resources under women's control are more likely to be allocated for beneficial purposes that promote family well-being than resources under men's control [67], which influence increased likelihood of access to health related services and/or education. In recent history, traditional family norms are evolving and new household orientations beyond male-dominated households are emerging as more common, with FHH becoming a more visible phenomenon globally [68]. According to Milazzo, Anamaria, and Dominique Van de Walle, the prevalence of FHH in Africa has grown and poverty among FHH has been declining at a faster rate since 1990. It is possible that previously poor FHH have leveraged new opportunities created by recent economic growth in Africa, as well as benefited from the expanded social protection in the region [69].

In our study, women with a parity of 2–4 births had lower odds of knowing their ovulatory cycle than women with a parity of one birth. There is evidence to suggest that poor ovulatory cycle knowledge and harmful social norms among older and high parity women in LMICs are possible explanations for this phenomenon [2, 70]. To improve ovulatory knowledge and prevent unintended pregnancy among those groups of women, communication resources for social and behavioral change that combine technical guidance with specific material for customers and providers must be considered [70].

### Implications of study findings

To the authors’ knowledge, no other study has investigated the association between health insurance coverage and knowledge of ovulatory cycle to inform policies or programs aimed at sexual and reproductive health (SRH), particularly family planning in LMICs. Findings from this work are new and have potential policy and program implications. The observed positive association between health insurance and ovulation knowledge supports the movement towards universal health coverage (UHC). Many LMICs have low uptake of modern contraception for a myriad of reasons ranging from lack of local stock, financial and cost barriers, and issues related to fear surrounding contraceptive use [18, 20]. This places many women, particularly adolescents, at higher risk of unmet need for family planning (i.e., unwanted or unplanned pregnancies) and associated negative health and socioeconomic inequities [71, 72]. As countries continue to work towards United Nations (UN) Sustainable Development Goal (SDG) 3.8—*achieving universal health coverage—*there is potential to contribute to increased SRH education, knowledge, and awareness among women, guiding reproductive decisions and reducing likelihood of unmet needs for family planning. Relatedly, previous work has found a positive association between women’s empowerment [40] as well as household decision making autonomy with coverage by health insurance [41] in Africa. Focusing on strategies and improving opportunities that contribute to women’s empowerment and autonomy may result in a positive effect on health coverage and knowledge of ovulation cycle. Additionally, SRH programs and approaches in SSA should target women who are in poorest quintiles, not formally educated, belong to male headed households, and have more than two births.

### Strengths and limitations

The association between health insurance and knowledge of the ovulatory cycle among women of reproductive age in 29 SSA countries was investigated using a large nationally representative sample. To the authors’ knowledge, no other study has investigated this association; findings have provided a basis to further investigate the pathways of how having health insurance in LMICs can work to protect against unintended/unplanned pregnancies in contexts where modern contraception is either not used/accepted or cannot be easily accessed. Nonetheless, some limitations need to be mentioned. First, because the study is cross-sectional, a causal-effect relationship cannot be established. Second, the DHS relied on self-reported data, which could be biased by recall. Third, contraceptive use or method was not included in the model knowing the low use of contraception in the region [18]. The focus was on the association between having health insurance and KOC, in an attempt to contribute to advancing the movement for universal health coverage while considering cultural norms/attitudes and availability of modern contraception. We acknowledge that this exclusion may limit the scope of our study and may not account for the full spectrum of factors influencing ovulatory cycle knowledge. Finally, due to data availability and constraints, we relied on surveys conducted at various points in the selected countries.

## Conclusion

The study found that knowledge of the ovulatory cycle was low among reproductive-age women in SSA, and health insurance had a positive relationship with knowledge of the ovulatory cycle. Additionally, women with higher education and wealth status as well as living in female-headed households were more likely to have knowledge of the ovulatory cycle, whereas parity (i.e., more than two births) had a negative association. Strategies for improving opportunities that contribute to women’s empowerment and autonomy as well as sexual and reproductive health approaches targeting women who are in poorest quintiles, not formally educated, belonging to male headed households, and having high parity should be considered.

## Data Availability

The datasets generated and/or analyzed during the current study are available in http://dhsprogram.com/data/available-datasets.cfm.
